# A fast and cost-effective method for apolipoprotein E isotyping as an alternative to *APOE* genotyping for patient screening and stratification

**DOI:** 10.1038/s41598-018-24320-3

**Published:** 2018-04-13

**Authors:** Olga Calero, Luis García-Albert, Andrés Rodríguez-Martín, Sergio Veiga, Miguel Calero

**Affiliations:** 1 Centro de Investigación Biomédica en Red sobre Enfermedades Neurodegenerativas (CIBERNED), Madrid, Spain; 2 Chronic Disease Programme (UFIEC)-CROSADIS, Instituto de Salud Carlos III, Madrid, Spain; 3 Biocross S.L, Valladolid, Spain; 4 CIEN Foundation-Queen Sofia Foundation, Instituto de Salud Carlos III, Madrid, Spain

## Abstract

Apolipoprotein E (apoE) is a 34 kDa glycoprotein involved in lipid metabolism. The human *APOE* gene encodes for three different apoE protein isoforms: E2, E3 and E4. The interest in apoE isoforms is high for epidemiological research, patient stratification and identification of those at increased risk for clinical trials and prevention. The isoform apoE4 is associated with increased risk for coronary heart and Alzheimer’s diseases. This paper describes a method for specifically detecting the apoE4 isoform from biological fluids by taking advantage of the capacity of apoE to bind “specifically” to polystyrene surfaces as capture and a specific anti-apoE4 monoclonal antibody as reporter. Our results indicate that the apoE-polystyrene binding interaction is highly stable, resistant to detergents and acid and basic washes. The methodology here described is accurate, easily implementable, fast and cost-effective. Although at present, our technique is unable to discriminate homozygous *APOE* ε4/ε4 from *APOE* ε3/ε4 and ε2/ε4 heterozygous, it opens new avenues for the development of inexpensive, yet effective, tests for the detection of apoE4 for patients’ stratification. Preliminary results indicated that this methodology is also adaptable into turbidimetric platforms, which make it a good candidate for clinical implementation through its translation to the clinical analysis routine.

## Introduction

Apolipoprotein E (apoE) is a 34 kDa glycoprotein involved in lipid metabolism^1^. The human *APOE* gene coding for this protein is polymorphic and encodes three apoE protein isoforms: E2, E3 and E4. These isoforms differ at the amino acid residues 112 and 158. Isoform E2 has cysteine residues at both sites, E4 has arginine residues at both sites, while E3, the most common form, has a cysteine at position 112 and an arginine at position 158^2,3^. The isoform E4 is associated with higher levels of cholesterol and increased risk for coronary heart and Alzheimer’s diseases (AD)^4,5^. In contrast, isoform E2 shows a protective effect against Alzheimer’s disease, but it is associated with familial type III hyperlipoproteinemia^6,7^. Thus, interest in *APOE* genotypes or apoE isoforms is high for epidemiological research, patient stratification and identification of those at increased risk of for clinical trials and prevention.

Several methods are commonly used for genotyping the three major *APOE* haplotypes. The most frequently used methods are: PCR-RFLP (Polymerase Chain Reaction-Restriction Fragment Length Polymorphism)^8,9^, capillary electrophoresis^10^, PCR plus sequencing or mass spectrometry^11^, ARMS-PCR (Amplification Refractory Mutation System-PCR)^12–14^, and SSP-PCR (Simple Sequence Specific Primer-PCR)^15^, RT-PCR (Real Time-PCR) detection by fluorescence melting curves^16^, FRET (Fluorescent Resonance Energy Transfer)^17,18^, allele specific RT-PCR^19^, and TaqMan probes^20^. All of these *APOE* gene-based methods are very effective, but require the procurement of informed consent for DNA extraction and analysis of genetic information, and cannot be easily implemented in the clinical analysis routine.

Mainly for research purposes, several alternative biochemical (non-genetic) methods are in use for the sensitive characterization of apoE isoforms from biological fluids such as plasma or CSF. The most commonly used are isoelectric focusing (IEF)-immunoblotting^21–24^ and sandwich ELISA, both in in-house^25^ and commercial assay (e.g. Biovision #K4699-100, MBL International #7635) setups. ELISA techniques use a pair of anti-apo-E antibodies (capture and reporter antibodies, being one of them specific for the E4 isoform), plus a secondary labeled-antibody for the sensitive detection. The main novelty of this method relies on the simplification of ELISA procedure, or other techniques such as turbidimetry, by exploiting the binding properties of apoE to polystyrene, precluding the use of a capture antibody or previous separation procedures, and allowing the sensitive detection of the apoE4 protein in diluted biological samples.

## Results

Preliminary studies of our group aiming at the characterization of the interaction of recombinant apoE with other proteins in an ELISA setup, indicated that even after blocking the polystyrene plates with suitable buffers, the apoE protein was able to bind with great efficiency to the plate.

**Figure 1:**
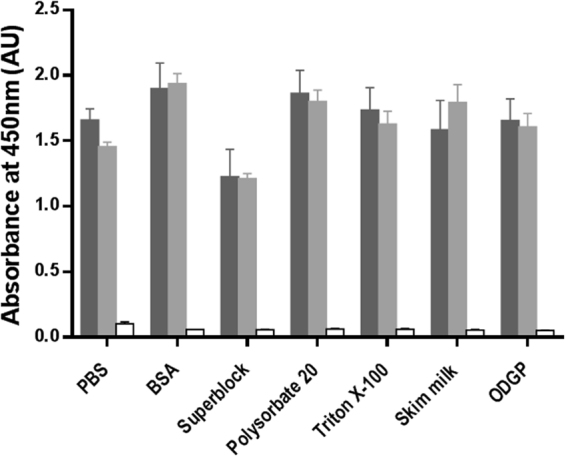
Binding of apoE4 to previously “blocked” ELISA plates. Recombinant apoE4 (dark gray bars), plasma from an *APOE* ε3/ε4 donor (light grey bars) or PBS (open bars) was allowed to interact with ELISA plate wells previously treated with different suitable blocking solutions: PBS (PBS with no blocking solution), BSA (0.25% BSA solution in 15 mM borate buffer containing 100 mM NaCl, pH 8.5), Superblock (Superblock T20 (ThermoFisher Scientific)), Polysorbate 20 (1% polysorbate 20 in PBS), Triton X-100 (1% Triton X-100 in PBS), Skim milk (5% skim milk in PBS containing 0.1% polysorbate 20), ODGP (8 mM Octyl b-D-glucopyranoside in PBS. Error bars represent the standard deviation of duplicated measures performed in two independent experiments.

As shown in Fig. 1, different buffers were ineffective blocking the binding of recombinant apoE4 to the plate as revealed by the use of the 4E4 antibody as reporter antibody.

These findings prompted us to explore whether this “specific” binding affinity of apoE to the polystyrene could be used as a potential capture method in substitution of an anti-apoE antibody for biological samples. Thus, we studied if the apoE contained in plasma samples was able to bind to the polystyrene plate. Interestingly, when we used a plasma sample from an *APOE* ε3/ε4 donor as the source of apoE4 and detected by the 4E4 antibody, we observed a similar binding to the plate “blocked” with different buffers (Fig. 1, light grey bars).

Similar experiments using recombinant apoE3 or plasma from *APOE* ε3/ε3 and ε2/ε3 individuals, and detected with the pan-apoE antibody showed equivalent results (data not shown), indicating that apoE binds to the polystyrene plate irrespectively of the isoform.

**Figure 2:**
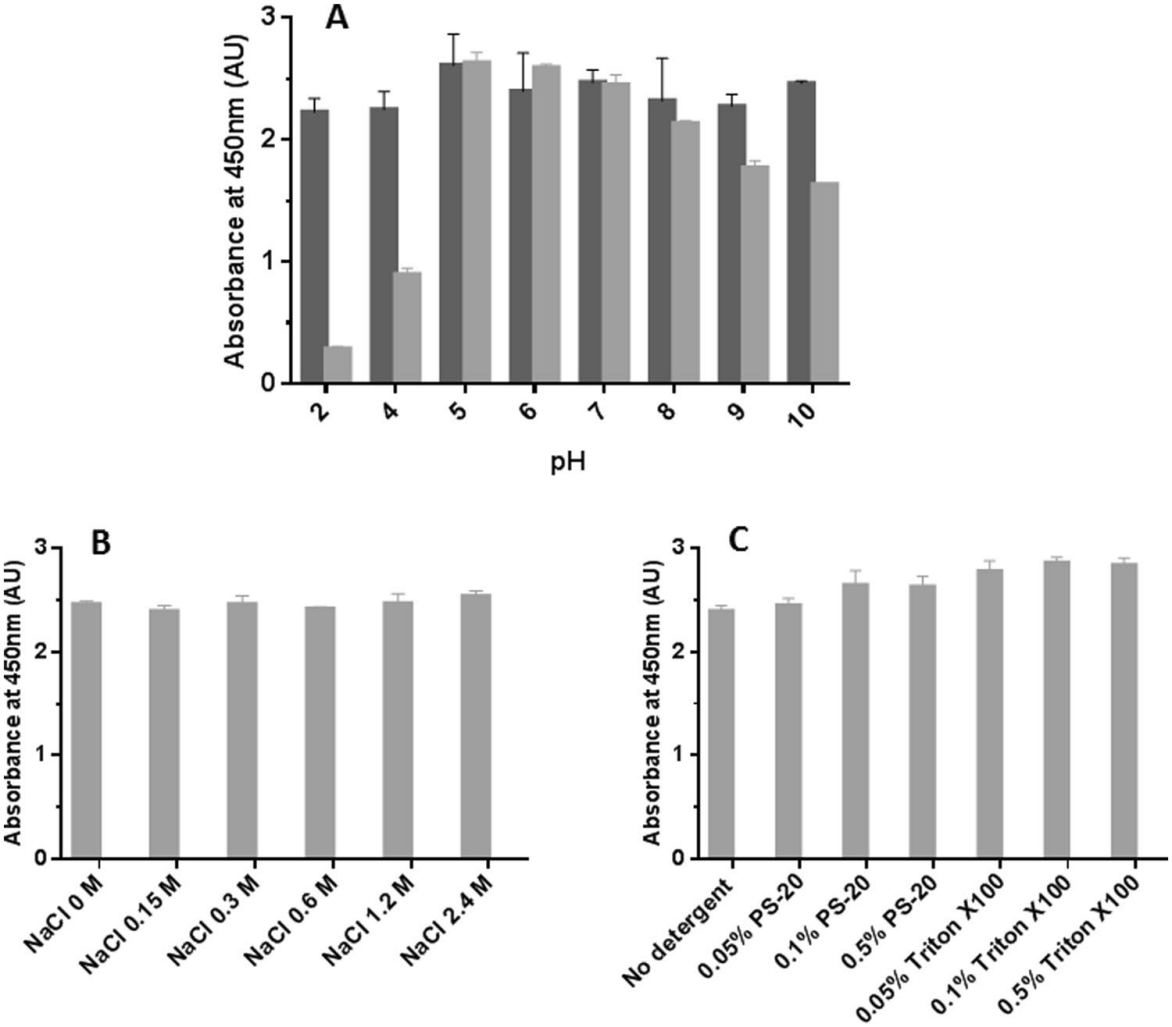
Stability of the binding of apoE to polystyrene as a function of pH, salt and detergent concentrations. Analysis of the stability of the binding of plasma apoE to polystyrene surfaces was challenged by varying pH (**A**), increasing salt concentrations (**B**) or detergent (0–0.5% polysorbate 20 or Triton X-100) (**C**) and detected by polyclonal pan-apoE antibody. Light and dark gray bars represent samples analyzed in wells blocked with a BSA-based or Superblock blocking solutions, respectively. Error bars represent the standard deviation of duplicated measures.

In order to characterize the nature and stability of the apoE-polystyrene binding, we studied the ability of different buffers and conditions to remove the plasma apoE already bound to the polystyrene plate by using the pan-apoE antibody as reporter. First, we explored the stability of the interaction apoE-polystyrene as a function of the pH in the range from pH 2 to 10, in wells blocked with either a BSA-based blocking solution or Superblock (Fig. 2A). We observed that the binding of apoE to the BSA-blocked polystyrene wells was fairly unaffected by the pH in the range studied. Interestingly, the binding of apoE to the Superblock-blocked polystyrene wells was unstable at pH below 5, suggesting that the presence of BSA may help to maintain a more stable microenvironment because of its own buffer capacity.

Next, we exposed the plasma apoE bound to BSA-blocked polystyrene ELISA plates to different concentrations of sodium chloride (0–2.4 M NaCl) in phosphate buffer pH 7.4 (Fig. 2B) or detergents in PBS (polysorbate 20 or Triton X-100, 0–0.5%) (Fig. 2C), followed by detection with the pan-apoE polyclonal antibody. Interestingly, we observed that the binding of apoE to the plate is stable in the presence of up to 2.4 M NaCl, and that the presence of moderate concentrations of detergents appears to enhance the detection of apoE compared to the control without detergent. These results suggest that the binding of apoE to the polystyrene plate is mediated by a combination of both electrostatic and hydrophobic forces.

**Figure 3:**
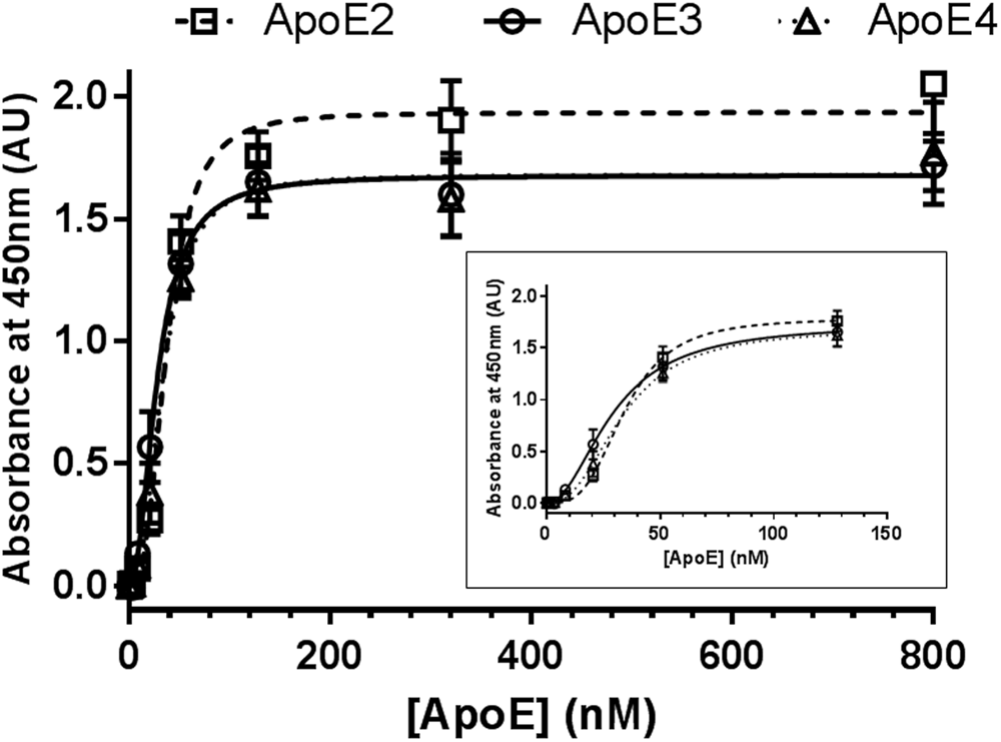
Saturation binding of apoE isoforms to the ELISA polystyrene surface. Variable concentrations (0–800 nM) of the three different recombinant apoE isoforms were added to ELISA plate wells, and incubated for 2 h at RT. After blocking, bound apoE was detected with a polyclonal pan-apoE antibody, followed by horseradish peroxidase-labelled anti-rabbit IgG. Error bars represent the standard deviation of duplicated measures. The graph shows a representative experiment of four independent tests performed by duplicate. The inset shows the same data represented from apoE concentrations in the 0–128 nM range.

To study the binding affinity of the interaction apoE-polystyrene, we estimated the dissociation constant (Kd) for the binding of each of the three isoforms of apoE to the polystyrene by saturation binding analysis under equilibrium conditions. For this purpose, in a polystyrene ELISA plate format, we performed a saturation binding assay by adding increasing concentrations of each recombinant apoE isoform to polystyrene wells, and then detected with pan-apoE polyclonal antibody. Interestingly, we have found that the binding of apoE to the polystyrene surface appears to be saturable and cooperative, indicated by a sigmoidal binding curve, rather than a hyperbola (Fig. 3). Fitting of the data to an one-site binding with Hill slope equation to account for cooperativity yielded Kd values from 22.3 to 32.6 nM and Hill slopes close to 2 for the binding of the different isoforms of apoE to the polystyrene (apoE2: Kd = 32.6 ± 8.3 nM, h = 2.07 ± 0.75; apoE3: Kd = 22.3 ± 5.5 nM, h = 2.14 ± 0.92; apoE4: Kd = 29.1 ± 10.1, h = 1.91 ± 0.54), with no statistically significant differences among the apoE isoforms (Fig. 3).

**Figure 4:**
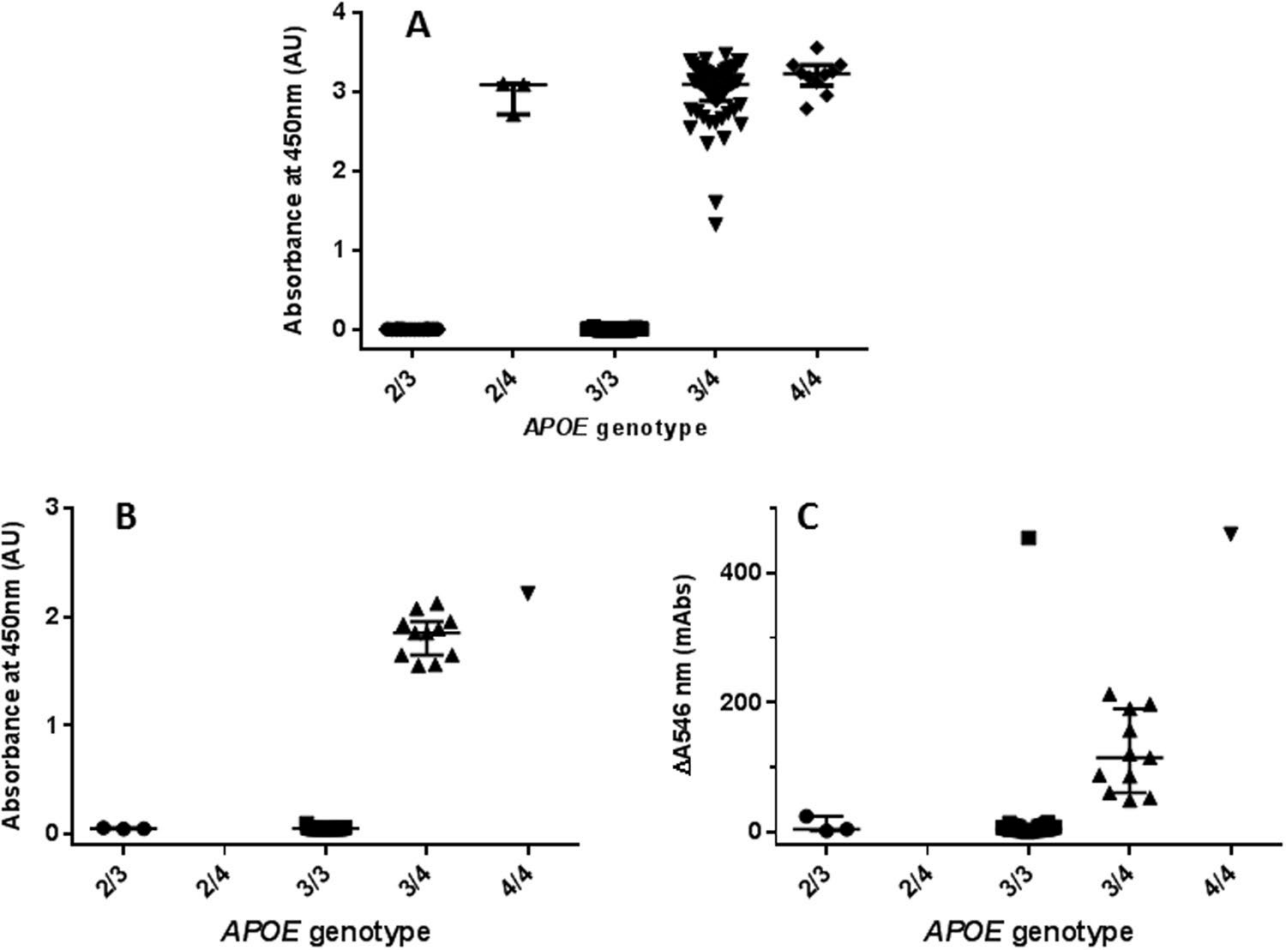
Analysis of the presence/absence of the apoE4 isoform in two cohorts of 230 (study cohort) and 50 (validation cohort) plasma samples by ELISA or by a turbidimetric assay based on LEIT. (**A**) Analysis of the presence of apoE4 in the study cohort of 230 plasma samples by ELISA setup stratified by *APOE* genotype. (**B**) Analysis of the presence of apoE4 in the validation cohort of 50 plasma samples by ELISA stratified by *APOE* genotype. (**C**) Analysis of the presence of apoE4 in the validation cohort of 50 samples by a turbidimetric assay stratified by *APOE* genotype. Error bars represent median with interquartile range.

The utility of this method (based on the binding of apoE to the polystyrene plate and its detection with an apoE4 specific antibody) for the analysis of the presence of the isoform apoE4 in biological samples was initially studied in a set of 230 human plasma samples (*study cohort*) from individuals previously genotyped by real-time PCR (ε2/ε3, n = 16; ε2/ε4, n = 4; ε3/ε3, n = 141; ε3/ε4, n = 59; ε4/ε4, n = 10). ELISA results revealed a 100% concordance with *APOE* genotypes (73 *APOE* ε4 carriers and 157 *APOE* ε4 non-carriers) (Fig. 4A).

Additionally, an equivalent analysis was performed in an independent cohort of 50 individuals (ε2/ε3, n = 3; ε3/ε3, n = 35; ε3/ε4, n = 11; ε4/ε4, n = 1) (*validation cohort*). In this replica cohort, we also observed total concordance of ELISA results with *APOE* genotype data (12 *APOE* ε4 carriers and 38 *APOE* ε4 non-carriers) (Fig. 4B).

In order to explore the potential translation of this methodology to other platforms, we tested the binding of recombinant apoE4 to 5.6 µm polystyrene beads in a Luminex platform. Binding of increasing concentrations of recombinant apoE4 to the beads yielded a saturation curve (data not shown) similar to the one found in ELISA, suggesting that the method could be applied to any polystyrene surface.

Next, we explored the replica cohort of 50 samples by a turbidimetric assay based on Latex-Enhanced Immunoturbidimetry Technology (LEIT) that relies also on the use of polystyrene beads. Preliminary analysis of this set of samples yielded 100% sensitivity (12/12 of *APOE* ε4 carriers) and 97.4% specificity (37/38 of *APOE* ε4 non-carriers) (Fig. 4C).

## Discussion

Based on previous experiments that indicated that recombinant and plasma apoE were able to bind to blocked polystyrene plates, we explored the properties and the potential utility of this interaction to determine the presence of the isoform apoE4 in plasma samples.

According to our results, the apoE-polystyrene binding interaction appears to be cooperative with Hill slopes around 2, suggestive of the binding of apoE oligomers to one polystyrene site. Within the range of concentrations used for the analysis of the apoE-polystyrene interaction, apoE is found to form dimers and tetramers. According to Garai and Frieden, monomeric and dimeric species are more prevalent in ApoE3 preparations, followed by ApoE4 and ApoE2^26^. For apoE4, they demonstrate that monomers predominate at ApoE concentrations less than 100 nM, while tetramers predominant above 2.5 μM, while the dimer population reaches maximum around 100 nM^26^. The cooperative binding observed for the apoE-polystyrene interaction, together with the facts on apoE oligomerization suggest that apoE dimers and tetramers present an increased affinity compared to apoE monomers; thus, the binding of one molecule of apoE to polystyrene favors the binding of further molecules to form dimers or tetramers. In average, our data indicate that the different apoE isoforms have a saturable “specific” interaction within the nanomolar affinity range showing minimum differences among apoE isoforms. In this study, ligand depletion effects cannot be completely ruled-out; but if present, they will lead to the underestimation of the Kd values, underscoring the relevance of this interaction.

The binding of apoE to polystyrene appears to be relatively independent of the apoE isoform, and highly stable, being resistant to detergents, acid and basic washes. The stability of this interaction prompted us to ponder about the potential implementation of immunoassays that under stringent conditions may allow the specific detection of the apoE4 isoform in biological samples. Exploiting these properties, we have developed a simple, fast, cost-effective and reliable method for apoE4 isotyping.

Our results in an ELISA format for two different cohorts of 230 and 50 plasma samples indicated 100% concordance with *APOE* genotypes for the detection of *APOE* ε4 carriers vs. non-carriers (Fig. 3A and B). However, although plasma samples from homozygous *APOE* ε4/ε4 tend to have higher absorbance readings than *APOE* ε3/ε4 and ε2/ε4 heterozygous, this specific ELISA setup appears to be unable to accurately discriminate *APOE* ε4 homozygous from heterozygous.

Interestingly, we observed similar results for the binding to polystyrene of other apolipoproteins, namely apoAI, apoCI, apoCIII and clusterin (data not shown). Therefore, potentially, similar protocols could be applicable for the detection of other apolipoproteins, precluding the need of additional purification, fractionation or concentration procedures, and allowing the specific and sensitive detection of target proteins.

At present, we are working in the adaptation, validation and implementation of the methodology here described to clinical chemistry turbidimetric platforms. Turbidimetric assays based on LEIT rely on the use of polystyrene beads coated with suitable antibodies. Then, agglutination is spectrophotometrically measured, giving a readout proportional to the concentration of the analyte of interest. Although LEIT is less sensitive than ELISA, it offers great advantages for relatively high concentrated analytes such as apolipoproteins, by allowing running tests in 5–20 minutes, and in fully-automated random access common clinical chemistry analyzers. The higher cost in raw material used by LEIT *vs*. ELISA is fully compensated by time saving, easiness and usability of the technology in hospitals laboratories and health service providers.

Preliminary results from the analysis of 50 plasma samples by LEIT showed encouraging results, indicating that turbidimetry signals were concordant with *APOE* genotypes in all cases, except for one false positive ε3/ε3 sample (Fig. 4C). Investigations are currently in progress to find out the cause of this false-positive discrepancy, and to maximize the discrimination of samples from *APOE* ε4 carriers vs. *APOE* ε4 non-carriers. Thus, the development here presented set the basis to adapt this methodology to turbidimetric technology for the detection of apoE4 in biological samples in clinical routine. ApoE4 detection by immunoturbidimetry overcomes many of the drawbacks and shortcomings of *APOE* gene analysis within clinical settings. First, our technology is not genetically based and can be performed in the same sample where other clinical routine analyses can be performed, without any further processing of the sample to extract the DNA or the requirement of an informed consent. Secondly, there is a significant saving in time, since our methodology can be used in high-throughput clinical chemistry platforms, allowing the analysis of hundreds of samples in a short period. Furthermore, *APOE* gene analysis by PCR is a method more complex and more expensive than the techniques normally used in a clinical laboratory setting. Additionally, *APOE* gene analysis is not included in the clinical routine and it is usually outsourced, which produce delays in reaching the results.

Although the method does not involve nucleic acid analysis or techniques alike, it provides information that can be correlated with individual genetic information, and it may perceive as a barrier for its implementation. However, it is important to note that very recently, the AD genetic test by 23andMe (https://www.23andme.com), which includes *APOE* genotype analysis, has attained FDA approval. The fact that our apoE isotyping method does not require the isolation and analysis of DNA simplifies the requirements for analysis (e.g. informed consent), being potentially applicable to different mostly acellular fluids such as serum, CSF, urine or saliva. At present, the main drawback of this isotyping technique is related to its inability to discriminate homozygous *APOE* ε4/ε4 from *APOE* ε3/ε4 and ε2/ε4 heterozygous. Future developments with isoform-specific antibodies for apoE3 and apoE2 (not currently commercially available) may help to overcome this limitation by providing a full isotype characterization, which may deliver analogous information to *APOE* genotyping.

Additionally, other approved turbidimetric tests (for example, those that detect proteins involved in the complement pathway such as factor D, C1 inhibitor, C3) can also indirectly provide genetic information, and they are widely used in clinical settings. Thus, we envisage that an apoE4 blood marker assay based on this development could be easily incorporated into routine dementia test profiles, allowing a fast identification of *APOE* ε4 carriers in the clinic for patient stratification and identification of those at increased risk of AD and cardiovascular disorders, as well as clinical trial enrichment. Moreover, it has been suggested that reducing the prevalence of modifiable risk factors of AD (hypertension, diabetes and hypercholesterolemia) would lower the risk, delay the onset and reduce the duration of AD^20^. Detection of patients at risk (i.e. *APOE* ε4 carriers) would allow applying lifestyle preventive interventions that could prevent or delay the development of the disease, which in turn could represent a significant saving for health care systems.

## Materials and Methods

### Plasma samples and *APOE* Genotyping

Plasma samples were collected from two different studies. An initial cohort (study cohort) was established from 230 individuals within the context of the project entitled “Validation trial for a multi-parameter diagnostic blood test for the diagnosis of Alzheimer’s disease” (Code: RH-VAL-2013-01). This study was approved by the Bioethics and Animal Welfare Committee from the La Paz Hospital (Madrid, Spain) as primary evaluation center (PI-1538); as well as by the Ethical Research Committees from different recruitment centers involved in the project.

Plasma samples used for the validation study and on the adaptation of the assay to turbidimetry were collected from 50 individuals within the context of the project entitled “Study for the development of a non-genetic test in blood for the identification of carriers of the ε4 alelle of the *APOE* gene (*APOE* ε4)” (Code: BCR-2017-01). The study was approved by the Clinical Investigation Ethical Committee from the Santa Creu I Sant Pau Hospital (Barcelona, Spain).

All samples were obtained after an informed consent form was signed by subjects, family members or legal guardians, as appropriate. All the data were analyzed anonymously, and clinical investigations have been conducted according to the principles expressed in the Declaration of Helsinki.

DNA samples isolated from peripheral blood from the 230 individuals of the study cohort (RH-VAL-2013 study) and 50 individuals for the validation cohort (BCR-2017-01 study) were used to determine the *APOE* haplotype for each sample according to a described method^18^.

### Polystyrene based detection of apoE4 by ELISA

For the specific detection of apoE4 by ELISA, we used 96-well plates (Nunc Maxisorb flat-bottom 96 well plate) blocked with a BSA-based blocking solution (0.25% BSA solution in 15 mM borate buffer containing 0.05% polysorbate 20 and 100 mM NaCl, pH 8.5). Alternatively, Superblock T20 blocking buffer from ThermoFisher Scientific has been used in certain experimental setups at room temperature (RT) for 16 hours. After blocking, the plates were washed four times with TBS 0.1% polysorbate 20 (TBS-T), and then incubated with diluted plasma 1:200 in TBS-T for 1 hour at RT. After washing, mouse monoclonal antibody specific for apoE4 (clone 4E4, Novus Biologicals, #NBP1-49529) diluted 1:2,000 in TBS-T plus 20% blocking solution, was added onto the wells and incubated for 45 minutes at RT. Then, the plates were washed and anti-mouse IgG-peroxidase (1:10,000 in TBS-T plus 20% blocking solution, USBiologicals, IgG, H&L (X-Adsorbed HRP #I1904-06) was added for 30 minutes at RT. After washing, in order to visualize the antibody binding, TMB chromogenic substrate (BioRad, #1721066) was used for 15 minutes at RT and measured, after stop with 2.15 N sulfuric acid, using a spectrophotometer at 450 nm with reference at 750 nm.

### Polystyrene-based detection of total apoE by ELISA

For the detection of total apoE in ELISA, the protocol was performed as above described, by using a pan α-apoE rabbit polyclonal antibody (Santa Cruz Biotechnology #sc-98573 H-223) as reporter antibody at 0.2 µg/ml, instead of the 4E4 monoclonal antibody, and a secondary anti-rabbit antibody IgG-peroxidase conjugated (US Biologicals #I1904-40A, IgG, H&L X-Adsorbed, (HRP) 1:10,000) as secondary antibody.

### Test of different blocking solutions

To test the binding of apoE to blocked polystyrene ELISA plates, recombinant apoE4 or plasma from an *APOE* ε3/ε4 donor were allowed to interact with ELISA plate wells previously treated with different blocking solutions. In brief, 200 microliters of blocking solutions were placed per well: (a) PBS (no blocking solution), (b) BSA-based blocking solution: 0.25% BSA solution in 15 mM borate buffer containing 0.05% polysorbate 20 and 100 mM NaCl, pH 8.5, (c) Superblock (TBS) (Thermo Fisher Scientific #37515) containing 0.1% polysorbate 20 (Superblock-T), (d) 1% polysorbate 20 in PBS, (e) 1%Triton X-100 in PBS, (f) 5% skim milk in PBS containing 0,1% polysorbate 20 and (g) 8 nM Octyl b-D-glucopyranoside (Sigma-Aldrich #O8001) in PBS containing 1.17% BSA. These blocking solutions were incubated for 16 h at RT, and then washed before proceeding with the ELISA assay with either the apoE4 specific monoclonal antibody 4E4, or the polyclonal pan-apoE antibody as above described.

### ApoE-polystyrene binding stability

The stability of the binding of plasma apoE to polystyrene surfaces was challenged by different pH (2–10) using a universal pH buffer system^27^, buffers containing increasing concentration of salt (0–2.4 M NaCl) or detergents (0–0.5% polysorbate 20 or Triton X-100). For this purpose, after binding to the plate of the apoE present in plasma (1:200 dilution), the wells were incubated for 1 hour at RT with the buffers of interest, detected with the pan-apoE polyclonal antibody, and then the assay continued as described above.

### Binding affinity of the interaction apoE-polystyrene

In order to calculate the dissociation constant^28^ for the apoE-polystyrene binding, we employed a ELISA format similar to the one described above using the three apoE isoforms (Abcam, recombinant human proteins: apoE2 #ab50244, apoE3 #ab50242 and apoE4 #ab50243) and the polyclonal pan-apoE antibody. Briefly, to non-blocked Nunc Maxisorb flat-bottom 96 well plates, we added decreasing half serial dilutions from 800 to 0.08 nM of recombinant apoE2, apoE3 and apoE4 diluted in TBS containing 0.1% polysorbate 20 (TBS-T) for 2 hours at RT. After washing with TBS-T, the plates were incubated with a BSA-based blocking solution (0.25% BSA solution in 15 mM borate buffer containing 0.05% polysorbate 20 and 100 mM NaCl, pH 8.5) for 1 hour at RT and washed again. The amount of bound apoE was determined by detection with rabbit polyclonal pan-apoE antibody (H-223 from SantaCruz at 1:1,000 dilution in TBS containing 20% of blocking solution for 1 hour at RT), and after washing followed by incubation with anti-rabbit-IgG-HRP secondary antibody (US Biologicals #I1904-40A at 1:10,000 dilution) for 30 min at RT. The assay was developed with TMB, stop after 10 min incubation and read at 450 nm.

The data were fitted to a saturation binding with Hill slope to account for cooperativity (“One site - Specific binding with Hill slope” in GraphPad Prism Software)^29^. The binding experiments were performed four times with duplicate measures. The Kd and Hill slope values are calculated as the mean ± standard deviation of the four experiments. In Fig. 3 is depicted one representative experiment.

### Luminex assay

To explore the binding of apoE to other polystyrene surfaces, we developed a Luminex assay using 5.6 µm polystyrene magnetic microspheres (MagPlex beads). Polystyrene beads, conserved in storage buffer (0.1% BSA and 0.02% polysorbate 20 in PBS, pH 7.4), were washed with PBS containing 0.05% polysorbate 20, and then incubated with increasing concentrations of recombinant apoE4 (0–100 nM) in PBS containing 0.05% polysorbate 20 for 1 hour at RT. Bound apoE4 was detected by monoclonal 4E4 antibody, and the protocol continued by using a phycoerythrin-labeled secondary antibody against mouse antibodies. Beads were analyzed using Luminex 200 system.

### Turbidimetric Assay based on Latex-Enhanced Immunoturbidimetry Technology (LEIT)

For the preliminary analysis of the presence of the apoE4 isoform in biological samples from a cohort of 50 samples by a turbidimetric assay based on LEIT, we adapted the ELISA setup to a turbidimetry-based kit. The kit consisted in two main reagents: R1 (which contains a highly specific anti-apoE4 antibody) and R2 (a solution containing the so called 200 nm “latex” (polystyrene) beads). During the assay, the anti-apoE4 antibody induces the agglutination of latex beads in the presence of apoE4 in the sample. This agglutination can be measured spectrophotometrically and used to discriminate *APOE* ε4 carriers from *APOE* ε4 non-carriers.

## References

[1] Mahley RW (1988). Apolipoprotein E: cholesterol transport protein with expanding role in cell biology. Science.

[2] Utermann G, Langenbeck U, Beisiegel U, Weber W (1980). Genetics of the apolipoprotein E-system in man. Am. J. Hum. Genet..

[3] Emi M (1988). Genotyping and sequence analysis of apolipoprotein E isoforms. Genomics.

[4] Mayeux R (1998). Utility of the apolipoprotein E genotype in the diagnosis of Alzheimer’s disease. Alzheimer’s Disease Centers Consortium on Apolipoprotein E and Alzheimer’s Disease. N. Engl. J. Med..

[5] Lahoz C (2001). Apolipoprotein E genotype and cardiovascular disease in the Framingham Heart Study. Atherosclerosis.

[6] Breslow JL (1982). Studies of familial type III hyperlipoproteinemia using as a genetic marker the apoE phenotype E2/2. J. Lipid Res..

[7] Corredor-Andrés, B. *et al*. Nephrotic syndrome associated with severe hypertriglyceridemia in a pediatric patient: Clinical Quiz. *Pediatr. Nephrol*. (In Press). 10.1007/s00467-018-3894-6 29532231

[8] Hixson JE, Vernier DT (1990). Restriction isotyping of human apolipoprotein E by gene amplification and cleavage with HhaI. J. Lipid Res..

[9] Zivelin A (1997). Improved Method for Genotyping Apolipoprotein E Polymorphisms by a PCR-Based Assay Simultaneously Utilizing Two Distinct Restriction Enzymes. Clin. Chem..

[10] Somsen GW (2002). Capillary electrophoresis with laser-induced fluorescence detection for fast and reliable apolipoprotein E genotyping. J. Chromatogr. B Analyt. Technol. Biomed. Life. Sci..

[11] Srinivasan JR (1998). Genotyping of apolipoprotein E by matrix-assisted laser desorption/ionization time-of-flight mass spectrometry. Rapid Commun. Mass Spectrom. RCM.

[12] Wenham PR, Newton CR, Price WH (1991). Analysis of apolipoprotein E genotypes by the Amplification Refractory Mutation System. Clin. Chem..

[13] Donohoe GG, Salomäki A, Lehtimäki T, Pulkki K, Kairisto V (1999). Rapid identification of apolipoprotein E genotypes by multiplex amplification refractory mutation system PCR and capillary gel electrophoresis. Clin. Chem..

[14] Yang YG (2007). Apolipoprotein E genotyping by multiplex tetra-primer amplification refractory mutation system PCR in single reaction tube. J. Biotechnol..

[15] Pantelidis P, Lambert-Hammill M, Wierzbicki AS (2003). Simple sequence-specific-primer-PCR method to identify the three main apolipoprotein E haplotypes. Clin. Chem..

[16] Papp AC, Pinsonneault JK, Cooke G, Sadée W (2003). Single nucleotide polymorphism genotyping using allele-specific PCR and fluorescence melting curves. BioTechniques.

[17] Nauck M, Hoffmann MM, Wieland H, März W (2000). Evaluation of the apo E genotyping kit on the LightCycler. Clin. Chem..

[18] Rihn BH (2009). APOE genotyping: comparison of three methods. Clin. Exp. Med..

[19] Calero O, Hortigüela R, Bullido MJ, Calero M (2009). Apolipoprotein E genotyping method by real time PCR, a fast and cost-effective alternative to the TaqMan and FRET assays. J. Neurosci. Methods.

[20] Koch W (2002). TaqMan systems for genotyping of disease-related polymorphisms present in the gene encoding apolipoprotein E. Clin. Chem. Lab. Med..

[21] Kataoka S, Paidi M, Howard BV (1994). Simplified isoelectric focusing/immunoblotting determination of apoprotein E phenotype. Clin. Chem..

[22] Eto M, Watanabe K, Moriyama T, Makino I (1990). Apolipoprotein E phenotyping from plasma by isoelectric focusing and immunoblotting. Tohoku J. Exp. Med..

[23] Steinmetz A (1987). Phenotyping of human apolipoprotein E from whole blood plasma by immunoblotting. J. Lipid Res..

[24] McDowell IF, Wisdom GB, Trimble ER (1989). Apolipoprotein E phenotype determined by agarose gel electrofocusing and immunoblotting. Clin. Chem..

[25] Uchida Y, Ito S, Nukina N (2000). Sandwich ELISA for the measurement of Apo-E4 levels in serum and the estimation of the allelic status of Apo-E4 isoforms. J. Clin. Lab. Anal..

[26] Garai K, Frieden C (2010). The association-dissociation behavior of the ApoE proteins: kinetic and equilibrium studies. Biochemistry (Mosc.).

[27] Ganesh K, Soumen R, Ravichandran Y, Janarthanan (2016). Dynamic approach to predict pH profiles of biologically relevant buffers. Biochem. Biophys. Rep..

[28] Pollard TD (2010). A Guide to Simple and Informative Binding Assays. Mol. Biol. Cell.

[29] Weiss JN (1997). The Hill equation revisited: uses and misuses. FASEB J..

